# Moralische Probleme der Versorgung von Menschen mit Demenz durch osteuropäische Live-in-Hilfen: eine ethische Analyse der Erwartungen von Angehörigen in Onlineforen

**DOI:** 10.1007/s00481-022-00708-8

**Published:** 2022-07-15

**Authors:** Simon Gerhards, Milena von Kutzleben, Mark Schweda

**Affiliations:** 1 Abteilung Ethik in der Medizin, Department für Versorgungsforschung, Carl von Ossietzky Universität Oldenburg, Ammerländer Heerstr. 114–118, 26129 Oldenburg, Deutschland; 2 Abteilung Organisationsbezogene Versorgungsforschung, Department für Versorgungsforschung, Carl von Ossietzky Universität Oldenburg, Oldenburg, Deutschland

**Keywords:** Live-in-Versorgung, Angehörige, Demenz, Migrierende Care-Arbeiter:innen, Laienmoral, Empirisch informierte Ethik, Live-in care, Family carer, Dementia, Migrant care worker, Lay moralities, Empirically informed ethics

## Abstract

In deutschen Privathaushalten sind derzeit schätzungsweise 100.000 bis 500.000 in Pendelmigration lebende Live-in-Hilfen tätig, viele von ihnen in der Versorgung von Menschen mit Demenz. Dabei deutet die primär sozialwissenschaftlich ausgerichtete Forschung zu Live-in-Versorgung auf vielfältige strukturell bedingte Missstände hin. Allerdings fehlt bislang eine eingehendere ethische Analyse und Erörterung mit Blick auf das Mikrosetting der betreffenden Versorgungsarrangements selbst.

Der vorliegende Artikel geht der Frage nach, welche moralischen Probleme und Konflikte im Mikrosetting häuslicher Live-in-Versorgung von Menschen mit Demenz auftreten und wie diese ethisch einzuschätzen sind. Zu diesem Zweck werden im Zuge einer qualitativen Inhaltsanalyse von 182 Beiträgen aus drei deutschsprachigen Onlineforen die Ansprüche betroffener Angehöriger von Menschen mit Demenz gegenüber Live-in-Hilfen herausgearbeitet und ethisch eingeordnet. Wie sich zeigt, werden diese als Dienstleistungserbringer:in, Pflegekraft, moralische:r Akteur:in und Familienmitglied adressiert.

In den vielfältigen und häufig enttäuschten Erwartungen der Angehörigen treten teils problematische und widersprüchliche Ansprüche gegenüber Live-in-Hilfen zu Tage. Eine ethische Analyse ihrer Legitimität und Kohärenz kann zur Verbesserung der individuellen Planung und Gestaltung, der institutionellen Organisation sowie der gesellschaftlichen Verständigung und rechtlichen Regulierung von Live-in-Versorgung beitragen.

## Abstract (en)

**Problem:**

An estimated 100,000–500,000 migrant care workers provide live-in care in German households, many of them caring for older people with dementia. Social research has identified a wide range of structural social problems associated with live-in care. However, a systematic ethical analysis and discussion is still missing.

**Arguments:**

This article explores the moral conflicts that arise in the microsetting of live-in arrangements for people with dementia. For this purpose, we conduct an ethical analysis of the expectations of relatives towards live-in care for people with dementia based on a qualitative content analysis of 182 contributions from three German-language online forums. These expectations address live-ins as service providers, professional nurses, moral agents, and family members.

**Conclusion:**

The diverse and often disappointed expectations of relatives are an expression of problematic and partly contradictory claims regarding live-in care for people with dementia. An ethical analysis of their legitimacy and coherence can help to improve the individual arrangement, the institutional organisation, and the moral framing and legal regulation of live-in care.

## Einleitung

Im August 2020 gab das Landesarbeitsgericht Berlin-Brandenburg der Klage einer Arbeitnehmerin aus Bulgarien statt. Die Klägerin hatte im Rahmen einer häuslichen 24-Stunden-Pflege eine hilfsbedürftige 96-Jährige betreut. Anstelle der vertraglich vereinbarten Arbeitszeit von 30 Stunden wöchentlich war sie allerdings nach eigenen Angaben meist von 6.00 Uhr morgens bis etwa 23.00 Uhr im Einsatz gewesen und hatte sich auch nachts bereithalten müssen. Das Gericht sprach ihr den geforderten Mindestlohn für eine tägliche Arbeitszeit von 21 Stunden zu und erklärte, die angesetzte Arbeitszeit sei für das vereinbarte Leistungsspektrum unrealistisch gewesen (LArbG Berlin-Brandenburg 21. Kammer 2020). Erst unlängst hat das Bundesarbeitsgericht das Urteil im Grundsatz bestätigt und klargestellt, dass ausländische Betreuungskräfte in deutschen Privathaushalten auch für die Bereitschaftszeit Anspruch auf Zahlung des gesetzlichen Mindestlohns haben (Bundesarbeitsgericht 2021).

Der Fall verdeutlicht die aktuelle Relevanz und zugleich das beträchtliche Konfliktpotenzial der häuslichen Versorgung durch – üblicherweise osteuropäische – Live-in-Hilfen, auch 24-Stunden-Hilfen genannt. Häufig ist eine demenzielle Erkrankung und der mit ihr einhergehende hohe Betreuungs- und Versorgungsaufwand Anlass zur Schaffung eines solchen Arrangements. Schätzungen zur Anzahl der in Deutschland tätigen Live-in-Hilfen in Pendelmigration reichen von 107.200 (Kantar 2019, S. 91) bis hin zu 500.000 Personen (Leiblfinger et al. 2020). Live-in-Versorgung wird sogar als „dritte Säule“ neben ambulanter Pflege und stationärer Langzeitpflege bezeichnet (VHBP e. V. 2022). Aktuelle Entwicklungen deuten darauf hin, dass diese Form der Versorgung auch zunehmend gesellschaftlich und politisch wahrgenommen wird. Die kürzlich veröffentlichte DIN SPEC 33454:2021-02 soll „Anforderungen und Mechanismen […] definieren, die allen Marktteilnehmern Orientierung bieten und bei erfolgreicher Anwendung zu einer höheren Versorgungsqualität, faireren Arbeitsbedingungen und zu mehr Transparenz führen“ (DIN 2021). Und laut eines vorab veröffentlichten Eckpunktepapiers des Bundesministeriums für Gesundheit stellt die Pflegereform 2021 eine neue staatliche Finanzierungsstruktur für Live-in-Hilfen in Aussicht, die auf der Umwandlung von Pflegesachleistungen in monetäre Hilfen zur Bezahlung von Live-in-Versorgung beruht (BMG 2020). Jüngst verspricht der Koalitionsvertrag der Ampelregierung eine „rechtssichere Grundlage für die 24-Stunden-Betreuung im familiären Bereich“ (SPD, Bündnis90/Die Grünen, FDP 2021, S. 81). Aus der primär sozialwissenschaftlich ausgerichteten Forschung zu Live-in-Versorgung ergeben sich allerdings zugleich vielfältige Hinweise auf erhebliche moralische Probleme und Konflikte, die einer ethischen Bestandsaufnahme und Auseinandersetzung bedürfen (Aulenbacher et al. 2021; Rossow 2021). So werden Live-In-Hilfen mitunter als „[m]oderne Sklavinnen“ (Engert 2014) bezeichnet und die von ihnen erbrachte Versorgung als „[m]enschenunwürdige Pflegearbeit in deutschen Privathaushalten“ (Emunds 2016) beschrieben.

Vor diesem Hintergrund gehen wir der Frage nach, welche moralischen Probleme und Konflikte sich im Mikrosetting häuslicher Live-in-Arrangements für Menschen mit Demenz ergeben und wie sie ethisch einzuordnen sind. Dazu wird eine ethische Untersuchung empirisch erhobener Erwartungen betroffener Angehöriger gegenüber Live-in-Versorgung vorgenommen. Die Grundlage bilden die Ergebnisse einer qualitativ-inhaltsanalytischen und systematisierend-ethischen Analyse von 182 Beiträgen aus drei Onlineforen, in denen sich Angehörige über Erfahrungen mit Live-in-Versorgung austauschen: chefkoch.de, Wegweiser Demenz, Deutsche Alzheimergesellschaft. Wir umreißen im Folgenden zunächst das ethische Problemfeld von Live-in-Versorgung für Menschen mit Demenz durch osteuropäische Haushaltshilfen und erläutern die Fragestellungen und Methoden unserer Untersuchung. Der Ergebnisteil bietet eine empirisch fundierte Typologie der Erwartungen von Angehörigen an Live-in-Versorgung und zeigt auf, dass die Live-in-Hilfen als Dienstleistungserbringer:in, als Pflegekraft, als moralische:r Akteur:in und als Familienmitglied adressiert werden. Die ethische Diskussion macht deutlich, dass in den vielfältigen und häufig enttäuschten Erwartungen der Angehörigen problematische und widersprüchliche Ansprüche gegenüber Live-in-Hilfen zu Tage treten. Eine ethische Analyse ihrer Legitimität und Kohärenz kann zur Verbesserung der individuellen Planung und Gestaltung, der institutionellen Organisation sowie der gesellschaftlichen Verständigung über und rechtlichen Regulierung von Live-in-Versorgung im Spannungsfeld persönlicher Anforderungen und struktureller Bedingungen beitragen.

## Hintergrund: Live-in-Versorgung für Menschen mit Demenz

Die wachsende Verbreitung von Live-in-Versorgung ist auf gesellschaftliche Entwicklungen und gesetzliche Rahmenbedingungen zurückzuführen (van Hooren et al. 2020). Der demographische Wandel und die zunehmende Erwerbstätigkeit von Frauen führen vor dem Hintergrund der subsidiär und familialistisch strukturierten Pflegeversicherung zu Versorgungslücken im häuslichen Versorgungssetting (Ehrlich 2019; Leitner 2013). Diese Lücken können für Menschen mit Demenz besonders ausgeprägt ausfallen (Hielscher et al. 2017). Ihre Versorgung ist mit besonderen Herausforderungen und Belastungen verbunden. Von den angehörigen Versorgungspersonen leisten 90 Prozent die Versorgungstätigkeiten an sieben Tagen die Woche und 80 Prozent auch nachts (Rothgang et al. 2015, S. 204). Die Inanspruchnahme von Live-in-Hilfen kann als ein Versuch der Kompensation dieser Versorgungslücken angesehen werden (Auth 2019; Klie 2014).

Etwa 400 Agenturen vermitteln in Deutschland Live-in-Hilfen aus dem meist osteuropäischen Ausland (Benazha et al. 2021; Leiber et al. 2019). Dabei werden diese sowohl im allgemeinen Sprachgebrauch (BMG 2020) als auch auf den Websites der Vermittlungsagenturen als „24-Stunden-Hilfen“ bezeichnet. Dieser Begriff deutet auf eine (arbeits-)rechtliche Problemlage hin (International Labor Organisation 2011). Live-in-Versorgung wird im politischen sowie rechts- und sozialwissenschaftlichen Diskurs als „Graubereich“ (Bucher 2018) oder „grauer Markt“ (Haberstumpf-Münchow 2020) bezeichnet. Dies ist Ausdruck einer Inkongruenz zwischen der Rechtslage und den Bedürfnissen vieler Angehöriger nach zeitlich umfassender häuslicher Versorgung. Es wird davon ausgegangen, dass 90 Prozent der Live-in-Hilfen informell beschäftigt werden (VHBP e. V. 2022; Rossow und Leiber 2019).

Die bisherige Forschung zu Live-in-Versorgung umfasst insbesondere sozial- und rechtswissenschaftliche Studien. Speziell die Praxis des „pass down of care“ (Hochschild 2001, S. 137), also der grenzübergreifenden Verlagerung von Sorgearbeit entlang intersektional verschränkter Diskriminierungsachsen wie gender/class/race/nation, stellt ein detailliert untersuchtes Phänomen dar. Es wird in seiner transnationalen Ausprägung als *Care-Chain-Phänomen* bezeichnet und nimmt einen zentralen Stellenwert in der Erforschung von Live-in-Versorgung durch migrierende Care-Arbeiter:innen ein (Lutz und Palenga-Möllenbeck 2011). Dabei werden transnationale Ungleichheiten und sozialethische Gerechtigkeitsfragen in den Vordergrund gerückt (Schilliger 2014; Hochschild 2003). Dagegen ist das Mikrosetting des häuslichen Live-in-Arrangements für Menschen mit Demenz vergleichsweise wenig erforscht. Zwar nimmt eine Reihe von kritischen sozialwissenschaftlichen Studien inzwischen die Perspektiven und Probleme der Live-in-Hilfen in den Blick (Aulenbacher et al. 2021; Schilliger 2014). Die Sichtweisen der übrigen beteiligten Personen und ihr Zusammenspiel in der Live-in-Versorgung wurden bislang allerdings nur vereinzelt untersucht (Rossow 2021; Horn et al. 2019). Eine systematische ethische Analyse steht nach wie vor aus (Städtler-Mach 2020).

## Methoden

Die vorgestellte Studie zielt im Sinne einer empirisch informierten Pflege- und Versorgungsethik auf eine empirische Exploration und ethische Analyse moralischer Problemfelder in der Live-in-Versorgung von Menschen mit Demenz. Zu diesem Zweck wurde eine qualitative Inhaltsanalyse von Beiträgen betroffener Angehöriger aus Onlineforen durchgeführt. Dieses Vorgehen lässt sich als „unobstrusive research“ (Salmons 2016) bezeichnen, da es zur Datenerhebung keiner Interaktion mit den Forumsnutzer:innen bedurfte. So ließen sich Einblicke in das aufgrund der oft problematischen arbeitsrechtlichen Verhältnisse sowie der Infektionsschutzmaßnahmen infolge der COVID-19-Pandemie schwer zugängliche Feld der Live-in Versorgung und erste Aufschlüsse über die hier bestehenden moralischen Probleme und Konflikte gewinnen.

Die Auswahl der Foren wurde auf der Grundlage einer ad hoc Online-Recherche nach Threads getroffen, die dem Austausch über die häusliche Versorgung von Menschen mit Demenz durch Live-in-Hilfen gewidmet waren. Als Suchbegriffe wurden in verschiedenen Kombinationen genutzt: „24“, „Stunden“, „Hilfe“, „Pflege“, „Polen“, „Osteuropa“, „Zuhause“, „Demenz“. Als Suchmaschine wurde Google verwendet und die Foren wurden gezielt mit der inurl-Suchfunktion durchsucht. Um die Verschiedenheit persönlicher Hintergründe, Informationsbedürfnisse und Kommunikationszusammenhänge zu berücksichtigen, wurden drei unterschiedliche Foren ausgewählt:

- Die Seite *wegweiser-demenz.de* wurde 2010 vom Bundesministerium für Familie, Senioren, Frauen und Jugend bereitgestellt und dient als Serviceportal mit Informationsangeboten zum Thema Demenz. Neben einem E‑Learning-Kurs und einer Adresssuche für Unterstützungsangebote gibt es thematisch unterschiedlich gelagerte „Ratgeberforen für Betroffene und Angehörige“, die von Sachverständigen moderiert werden.
- Die Foren der *Deutschen Alzheimer Gesellschaft* dienen als „Treffpunkt zum Erfahrungsaustausch für Betroffene und Interessierte“ (Deutsche Alzheimer Gesellschaft e. V. 2022). Die offenen Foren umfassen über 900 Threads mit ca. 4000 Beiträgen, die ersten von 2004. Die Moderation achtet auf die Einhaltung der Nutzungsbedingungen und bringt sich als sachkundige Instanz beratend in die Diskussionen ein.
- *Chefkoch.de* ist ein Webportal der Chefkoch GmbH mit Themenschwerpunkt Kochen und über 4,1 Mio. registrierten Nutzer:innen in Deutschland. Es umfasst auch Foren zu Themen ohne direkten Kochbezug. Moderator:innen werden aus der Gruppe der aktiven Nutzer:innen ernannt. Sie überwachen die Einhaltung der Forumsregeln, treten jedoch nicht als Sachverständige auf und schalten sich nicht beratend in die Diskussionen ein.

Es wurden mehrere Kriterien zum Einschluss von Threads in das Analysekorpus in Anschlag gebracht. Der thematische Fokus hatte auf der Versorgung von Menschen (mit Demenz) unter Einbeziehung migrierender Live-in-Hilfen zu liegen. Zudem sollten die Threads eine gewisse themenspezifische inhaltliche Dichte aufweisen, also einen ausreichenden Anteil themenbezogener Beiträge. Der aktuellste Beitrag eines Threads musste zudem zwischen 2012 und Mai 2020 liegen. Die Beiträge sollten in deutscher Sprache verfasst und der Zugang öffentlich und ohne Registrierung möglich sein. Um Beiträge kontextualisiert analysieren zu können, wurden nur ganze Threads eingeschlossen. Insgesamt wurden acht Threads mit insgesamt 182 Beiträgen von ca. 85 verschiedenen Nutzer:innen untersucht: zwei aus dem Forum von „Wegweiser Demenz“, vier aus dem der Deutschen Alzheimergesellschaft und zwei aus dem der Chefkoch-Community, Themenbereich „Familie und Kinder“.

Die qualitative Inhaltsanalyse orientierte sich an einer *inhaltlichen Strukturierung* (Kuckartz 2018), um themenspezifische Textstellen zu identifizieren und unter inhaltlichen Gesichtspunkten zusammenzufassen und zu ordnen. Die Analyse wurde softwaregestützt mit MAXQDA durchgeführt. Im Fokus standen manifeste und latente moralische Problemfelder. Die Kategorienbildung erfolgte sowohl theoriegeleitet deduktiv als auch induktiv am Material. Mit der inhaltlichen Strukturierung war eine ethische Analyse verknüpft, die auf ethische Einordnung und Reflexion der identifizierten moralischen Problemfelder abzielte und in die im Folgenden dargestellte Typologisierung mündete. Dabei wurden *individualethische* Aspekte durch tugendethische Analysekategorien erfasst. Sie erlauben eine tiefenscharfe Betrachtung der Bewertungen von Individuen als moralische Akteur:innen (Rippe und Schaber 1998, S. 11). Handlungen, die von Angehörigen in den Foren beschrieben wurden, konnten so auf moralische Annahmen zu motivationalen Strukturen und Charaktereigenschaften der Live-in-Hilfen bezogen werden. *Pflege- und versorgungsethische* Aspekte wurden in einem prinzipienethischen Bezugsrahmen (Beauchamp und Childress 2012) untersucht. Ursprünglich für die professionsethische Evaluation ärztlichen Handelns formuliert, finden die vier Prinzipien Respekt vor Autonomie, Benefizienz (Fürsorge/Wohltun), Non-Malefizienz (Nicht-Schaden) und Gerechtigkeit mittlerweile auch Anwendung in der Pflegeethik (Riedel 2016). Der Ansatz erschien angemessen, um moralische Bewertungen des Versorgungshandelns von Live-in-Hilfen zu analysieren, das häufig auch pflegerische Tätigkeiten umfasst (Schilliger 2014, S. 241). *Sozialethische Aspekte* wurden schließlich im Horizont einer kritischen Care-Ethik analysiert, die auch gesellschaftliche Bedingungen der Gestaltung von Sorgebeziehungen wie etwa patriarchale Machtstrukturen und globale Ausbeutungsverhältnisse in den Blick nimmt (Held 2006).

## Ergebnisse: Das Spektrum der Erwartungen Angehöriger an Live-in-Hilfen

In den analysierten Threads werden von Angehörigen verschiedene Gründe für die Erwägung der Schaffung eines Live-in-Arrangements genannt. Dazu zählt der Wunsch der versorgungsbedürftigen Person, weiterhin zu Hause zu wohnen, sowie der durch die demenzielle Erkrankung entstehende Bedarf an zeitlich umfassender, quasi kontinuierlicher Betreuung und Versorgung. Die räumlich-geographische Distanz von Angehörigen zur zu versorgenden Person, die Unvereinbarkeit der eigenen Erwerbstätigkeit mit der zeitintensiven Versorgung von Menschen mit Demenz und/oder der mit (antizipierter) Überforderung einhergehende Wunsch nach Entlastung motivieren dann die Suche nach praktikablen Lösungen. Da der durch die Pflegeversicherung abgedeckte Umfang ambulanter Versorgungsleistungen meist als unzureichend empfunden wird, erscheint die Inanspruchnahme vergleichsweise kostengünstiger Live-in-Versorgung vielfach naheliegend und geradezu alternativlos. Zugleich eröffnet sich ein breites Spektrum unterschiedlicher Erwartungen, die sich vonseiten der Angehörigen auf die Live-in-Hilfe richten. Diese Erwartungen lassen sich idealtypisch vier Rollen zuordnen, die im Folgenden eingehender im Hinblick auf ihren Kerngehalt, ihre inneren Ambivalenzen sowie ihre Spannungen und Widersprüche untereinander betrachtet werden. Es handelt sich um Erwartungen an die Live-in-Hilfe als Dienstleistungserbringer:in, als professionelle Pflegekraft, als moralische:r Akteur:in und als Familienmitglied.

### Die Live-in-Hilfe als Dienstleistungserbringer:in

Ein Großteil der Erwartungen Angehöriger richten sich an die Live-in-Hilfen als Dienstleistungserbringer:in. Sie sind charakterisiert durch ein Denken in vertraglichen Dimensionen. Ein Vertrag kommt durch die freiwillige, selbst gewählte Verpflichtung zur Erfüllung gegenseitiger Versprechen zustande. Die primären Verpflichtungen der Live-in-Hilfe als Dienstleistungserbringer:in bestehen demnach in der Einhaltung der Vereinbarung durch Erbringung der vertraglich vorgesehenen Leistungen.

Als Dienstleistungserbringer:in ist die Live-in-Hilfe Mittel zum Zweck. Sie wird bezahlt, um den Erhalt der Versorgungssituation in der eigenen Häuslichkeit zu gewährleisten, die Angehörigen zu entlasten und die höhere Lebensqualität der individuellen Versorgung in der gewohnten Umgebung zu garantieren (Chef 1.2, Pos. 56). Von ihren inneren Befindlichkeiten und privaten Problemen – den „persönliche[n] Päckchen“ (Alz 7.10, Pos. 14) – kann dabei abgesehen werden. Außerdem sind Live-in-Hilfen als Erbringer:innen von Versorgungsdienstleistungen auf einem globalisierten Care-Markt austauschbar und durch andere Live-ins ersetzbar. So werden Vermittlungsagenturen mit dem spontanen Umtausch von Hilfen beauftragt, wenn diese die an sie gestellten Erwartungen nicht erfüllen. Die Vermittlungsagentur, schreibt ein:e Forumsnutzer:in, reagiere „immer sofort, auch abends und am Wochenende, hat auch schon zu sofort [sic] ausgetauscht“ (Alz 1.22, Pos. 45).

Der Blick auf die Live-in-Hilfe als Dienstleister:in motiviert darüber hinaus ein Narrativ von Live-in-Versorgung als Win-Win-Situation für alle Beteiligten. Vor dem Hintergrund der sehr unterschiedlichen nationalen Lohnniveaus werden die Verdienstmöglichkeiten der Hilfe in der Pendelmigration hervorgehoben. Die ökonomischen Anreize und Motivationen der Live-in-Hilfen werden als Beweis für die Freiwilligkeit der Dienstleistungserbringung in Form von Rund-um-die-Uhr-Betreuung angeführt. In Berufung auf den kontraktualistischen und damit vermeintlich freiwilligen Charakter des Live-in-Verhältnisses werden so Aussagen relativiert, die die Arbeitsverhältnisse von Live-in-Hilfen als problematisch oder sogar ausbeuterisch kritisieren. Strukturelle transnationale Abhängigkeits- und Ungleichheitsverhältnisse werden vor dem Hintergrund einer freiwilligen Entscheidung zur Pendelmigration als weniger relevant dargestellt. Ein:e Nutzer:in betont z. B., dass „sie hierher kommen“ geschehe „wohl auf ihren eigenen Wunsch hin“ (Chef 2.X, Pos. 8). Diese Annahme wird dann zur Relativierung der Distanz zwischen Live-in-Hilfen und ihren eigenen Familien herangezogen. Der:die gleiche Nutzer:in bewertet die Aussage, „dass sie [die Live-in-Hilfen] von ihren Kindern getrennt werden“ als „etwas theatralisch“ (Chef 2.X, Pos. 8).

Die Rollenerwartungen an die Live-in-Hilfe als Dienstleistungserbringer:in konfligieren zugleich mit Vorstellungen, die eher einem familiären Verpflichtungsdenken entstammen. Einerseits werden Live-ins in einer kontraktualistisch anmutenden Argumentation mit Verweis auf ihre Bezahlung in die Pflicht genommen, umfassende Care-Dienstleistungen zu erbringen. Andererseits ist das Beschäftigungsverhältnis sowohl durch eine unzureichende Eingrenzung der Aufgabenbereiche charakterisiert als auch durch eine unscharfe Trennung von Arbeits- und Freizeiten, wie sie in familiären Versorgungsverhältnissen vorherrscht. Das Rollenverständnis der Live-in-Hilfe als Dienstleistungserbringer:in wird so dazu genutzt, einseitige Ansprüche an die Erfüllung der erwarteten Versorgungsleistungen zu stellen und dabei die eigentlich mit einem Vertrag einhergehenden Rechte der:s Arbeitnehmer:in zu vernachlässigen. So werden einerseits Erwartungen formuliert, die über vertraglich zulässige Anforderungen hinausgehen. Die Live-in-Hilfe solle „Freude bei der Arbeit“ (Alz 1.11, Pos. 33) haben und gleichzeitig mit hohem persönlichen Einsatz gänzlich für die Versorgung zur Verfügung stehen. Andererseits werden Effizienzdenken und Arbeitsverdichtung aufseiten der Live-in-Hilfe, die aus der Perspektive einer:s Dienstleistungserbringer:in durchaus angemessen erscheinen, von Angehörigen vor dem Hintergrund familiärer Rollenerwartungen als illegitim angesehen. Als „von der Haltung geprägt: Hauptsache sauber und satt“ werden Hilfen kritisiert, die nur das Nötigste an Arbeit tun (Alz 1.11, Pos. 33).

### Die Live-in-Hilfe als Pflegekraft

Häufig beziehen sich die Erwartungen der Angehörigen zudem auf die Professionalität der Live-in-Hilfe. Die durch sie geleistete Versorgung soll gewissen Standards fachlich begründeten und entwickelten Wissens und Könnens entsprechen. Als Leitbild und Maßstab scheint dabei die Vorstellung einer ausgebildeten Pflegekraft im Hintergrund zu stehen.

Tatsächlich wird der Schwerpunkt der Tätigkeit von Live-in-Hilfen ungeachtet ihrer häufigen Bezeichnung als Haushaltshilfe und der tatsächlichen beruflichen Qualifikation meist im pflegerischen Bereich gesehen. Dabei reichen ihre Aufgaben auch über die allgemeine Grundpflege hinaus in den Bereich der Behandlungspflege. Sie umfassen mithin nicht nur „allgemeine Pflege [...], also Körperpflege“ (Chef 2.4, Pos. 4), Helfen beim An- und Auskleiden, Wechseln von Inkontinenzmaterialien, Waschen und Duschen, „Ins Bett bringen“ (Alz 2.8, Pos. 14), „Aktivierung und Unterhaltung“ (Alz 1.13, Pos. 36), Haushalt, Aufräumen und Putzen, Kochen, Einkaufen, Bügeln, Erreichbarkeit und Bereitschaft – „in Notfällen eben auch nachts“ (Alz 2.11, Pos. 21), sondern etwa auch die Verabreichung von Medikamenten (Chef 2.4, Pos. 12).

Dabei werden auch spezifische Anforderungen der professionellen Pflege von Menschen mit Demenz in Anschlag gebracht, z. B. ein aktivierender Ansatz, ein kompetenter Umgang mit als herausfordernd wahrgenommenem Verhalten oder gar ein Eingehen auf neuropsychiatrische Besonderheiten bestimmter demenzieller Erkrankungen. Angesichts der hohen Belastungen, die mit der Pflege eines Menschen mit Demenz einhergehen können, wird den Live-ins in diesem Zusammenhang ausdrücklich eine „beruflich“ begründete „emotionale Distanz“ zugeschrieben (Chef 2.7, Pos. 12). Darüber hinaus werden auch Erwartungen hinsichtlich eines angemessenen Verhaltens formuliert, die an berufsethische Grundsätze erinnern. Insbesondere Respekt vor der Autonomie der Versorgten und Sorge für ihr Wohlergehen erscheinen in vielen Forumsbeiträgen als Anliegen. Ein:e Nutzer:in berichtet z. B. davon, er:sie habe versucht, der Live-in-Hilfe zu erklären, „dass man doch bitte auf die Bedürfnisse meiner Mutter eingehen solle“, das heißt „ihr ,Nein‘ zu respektieren“ (Alz 2.8, Pos. 14).

Gemessen an diesen professionellen Standards schneiden die Live-in-Hilfen im Urteil der Angehörigen allerdings oft schlecht ab: „Ausser Bevormundung […] und recht ungeschicktes Auftreten läuft da nicht viel“, schreibt ein:e Nutzer:in (Alz, anonym, Pos. 16). Vielfach werden ihnen mangelnde fachliche Kenntnisse und Fähigkeiten vorgehalten, insbesondere im Umgang mit Menschen mit Demenz. Mitunter wird dies auf charakterliche Schwächen, kulturelle Unterschiede in Haltung und Handeln oder eine vermeintlich rückständige Pflegepraxis im Herkunftsland zurückgeführt. Die Diskrepanz zwischen den hohen Anforderungen hinsichtlich der Versorgungsqualität auf der einen und der vergleichsweise niedrigen Bezahlung sowie den oft kaum angemessen geregelten Arbeitszeiten und -bedingungen auf der anderen Seite wird selten zur Kenntnis genommen und reflektiert. Stattdessen werden zur Untermauerung der erhobenen Ansprüche je nach Bedarf vertragliche Vereinbarungen, allgemeine Hilfsbereitschaft oder eine besondere persönliche Beziehung ins Spiel gebracht. Die Möglichkeit, einen ambulanten Pflegedienst zur professionellen Ergänzung der Live-in-Versorgung hinzuzuziehen, wird in den Foren durchaus diskutiert. In den Augen einiger Angehöriger würden die dadurch entstehenden Mehrkosten allerdings eher das Live-in-Arrangement als besonders günstige Versorgungsform ad absurdum führen. Vor diesem Hintergrund zeichnet sich hier eine spannungsreiche und letztlich paradoxe Konstellation von Erwartungen ab, in der eine hohen professionellen Maßstäben entsprechende Versorgungstätigkeit zum Preis und den Konditionen einer unqualifizierten Dienstleistung, einer altruistischen Hilfe oder gar eines unbezahlten Freundschafts- oder Liebesdienstes in Anspruch genommen werden soll.

### Die Live-in-Hilfe als moralische:r Akteur:in

Tatsächlich werden an die Live-in-Hilfe nicht nur Erwartungen gerichtet, die sich aus den besonderen vertraglichen Übereinkünften oder professionellen Aufgaben des Settings ergeben, sondern auch solche, die allgemeine moralische Ansprüche des Umgangs miteinander zum Ausdruck bringen. Sie adressieren die Hilfe in ihrer Eigenschaft als moralische:r Akteur:in und lassen sich ethisch im Sinne von Tugenden fassen.

Die entsprechenden Erwartungen beziehen sich unter anderem auf die Tugenden der Verlässlichkeit, Ehrlichkeit, Rücksichtnahme und Höflichkeit. Da die Live-in-Hilfe in den Haushalt eines kranken und nur eingeschränkt selbstständigen Menschen einzieht, sind Ehrlichkeit und Verlässlichkeit zentrale Erwartungen an sie. Der Wunsch der Angehörigen nach Entlastung geht einher mit der Erwartung, dass die übertragenen Aufgaben pflichtbewusst und zuverlässig verrichtet werden. Gerade im Kontext der Betreuung von Menschen mit Demenz spielt hier die verlässliche Übernahme von Verantwortung für die Sicherheit der zu versorgenden Person eine wichtige Rolle: Alz 1.4 wünscht sich entsprechend „eine verläßliche 24-Stunden-Präsenz durch eine Hilfe im Haus“ (Alz 1.4, Pos. 6). Unehrliche Aussagen der Live-in, etwa in Bezug auf die eigenen sprachlichen oder pflegerischen Kompetenzen, stellen diese Vertrauensbasis in Frage und werden deshalb als gefährdend für Stabilität und Sicherheit des Live-in-Settings verurteilt. Unzuverlässigkeit in der Versorgung ist aus Sicht der Angehörigen nicht tolerierbar und führt häufig zur Entlassung und der Suche nach einer anderen Hilfe.

Als weitere Tugenden im Zusammenleben werden Höflichkeit und Rücksichtnahme auf die Bedürfnisse der zu versorgenden Person hervorgehoben. Gewissermaßen als Gast in der Wohnung der zu versorgenden Person wohnend und arbeitend, wird von der Live-in-Hilfe erwartet, dass sie ihr Verhalten an den dort geltenden Regeln und Werten ausrichtet. Die Deutungshoheit über höfliches und unhöfliches Verhalten obliegt den Gastgebenden. Aus diesem Grund können interkulturelle und intergenerationelle Unterschiede zu Konflikten über (un-)angemessenes Verhalten führen. So wird z. B. das Verhalten, „[p]ausenlos zu telefonieren egal ob irgendwer im Raum war oder nicht, – als ob eben niemand mit im Raum war“, von einer Angehörigen kritisiert und vor dem Hintergrund der Irritation ihrer zu versorgenden Mutter als „unverschämt und ungezogen“ bezeichnet (Alz 2.8, Pos. 15). Die Rücksicht auf die Bedürfnisse anderer steht auch hier im Vordergrund. Dies zeigt sich auch daran, dass Angehörige es mitunter als belastend empfinden, wenn die Live-in-Hilfen von persönlichen Sorgen erzählen. Die herausfordernde Versorgungssituation eines Menschen mit Demenz solle nicht noch zusätzlich durch Erzählung von Schicksalsschlägen, finanziellen und privaten Notlagen belastet werden, so die Erwartungshaltung.

Wie bereits angeklungen, werden Erwartungen an das tugendhafte Verhalten der Live-in-Hilfen immer wieder enttäuscht. So wird der zu versorgende Mensch mit Demenz mitunter durch unzuverlässiges oder unzureichend verantwortungsbewusstes Verhalten der Hilfe gefährdet. Eine Live-in-Hilfe lässt etwa ein „brennendes Bügeleisen stehen während sie das Haus verlässt“ oder lässt „fremde Leute ins Haus und allein mit meiner Oma ohne diese zu kennen“, so die Kritik in den Onlineforen (Alz 1.21, Pos. 45). In diesem Kontext wird auch von Live-ins berichtet, die aufgrund von Alkoholmissbrauch im Versorgungssetting als unzureichend zuverlässig und vertrauenswürdig angesehen wurden. Eine Hilfe habe versucht „sich betrunken […] an meiner 95-jährigen Großmutter hochzuziehen“ (Alz 1.21, Pos. 45). Angehörige kritisieren außerdem explizit gewaltsames Verhalten von Live-in-Hilfen gegenüber den ihnen anvertrauten Menschen mit Demenz: Die zu versorgende Person wurde „gewaltsam in den Stuhl“ (Alz 2.8, Pos. 14) gedrückt oder „ins Badezimmer geschubst, um ihn zu rasieren“ (Alz 2.12, Pos. 23). Die durch das gewaltsame Verhalten einer Live-in-Hilfe entstandenen Verletzungen seien von dieser quittiert worden mit: „Alte Leute oft blaue Flecken. Normal“ (Alz 2.12, Pos. 23).

Die Erwartungen an die Live-in-Hilfe als moralische:r Akteur:in erscheinen nicht nur im Nebeneinander mit anderen Rollenerwartungen als widersprüchlich. Auch grundsätzlich wird ihre Angemessenheit gelegentlich in Frage gestellt. So können die persönlichen Problemsituationen von Live-in-Hilfen von Angehörigen auch im Sinne der Einschränkung allgemeiner moralischer Erwartungen im Live-in-Setting gleichsam als moralisch mildernde Umstände angesehen werden. Wenn z. B. persönliche Not und finanzielle Bedürftigkeit die Gründe für die Arbeit als Live-in-Hilfe darstellen, erscheint unehrliches Verhalten in Form von Falschangaben in Lebensläufen als ein aus der Not geborenes und damit eher entschuldbares Verhalten. Die Berichte von Alkoholismus verstärken den Eindruck, dass Live-in-Hilfen unter hohen psychischen Belastungen stehen und die Pendelmigration mitunter als Ausweg aus persönlichen Belastungssituationen in der Heimat wählen. Entsprechend schreibt eine Angehörige, dass sie Live-in-Hilfen häufig als Frauen erlebt habe, „die aus einer besonderen Notsituation diese Arbeit wählten“, die „gescheitert waren und nirgendwo mehr einen Fuß auf den Boden bekamen“ (Alz, anonym, Pos. 13).

### Die Live-in-Hilfe als Familienmitglied

Wie bereits anklang, richten sich schließlich auch Erwartungen an die Live-in-Hilfen, die eher an familiäre Rollen und Verantwortlichkeiten und damit an Ansprüche gegenüber nahen Verwandten erinnern. Diese Erwartungen an die Hilfe als Familienmitglied werden allerdings kaum ausdrücklich formuliert. Sie kommen überwiegend indirekt zum Ausdruck, vor allem in Vorstellungen einer emotionalen oder relationalen Motivation bzw. Qualität des Versorgungshandelns.

Tatsächlich wird von der Live-in-Hilfe oft wie selbstverständlich eine selbst- und grenzenlose Übernahme von Versorgungsverantwortung erwartet, die weder im Rahmen professioneller Zuständigkeiten noch vertraglich vereinbarter Dienstleistungen angemessen erschiene. Gerade die Vorstellung einer besonderen intrinsischen Motivation und überschießenden Einsatzbereitschaft in der Versorgung aus einem persönlichen Zugetansein und einer gefühlsmäßigen Verbindung mit der gepflegten Person und ihren Angehörigen heraus weist in eine solche Richtung. Live-in-Versorgung geht über praktische Verrichtungen hinaus und ist Beziehungsarbeit, bei der „die Chemie“ stimmen muss (Chef 1.5, Pos. 21; Chef 1.2, Pos. 6). Entsprechend werden auch mit Blick auf den konkreten Vollzug des Versorgungshandelns verschiedentlich besondere emotionale Qualitäten wie „liebevoll“ (Alz. 1.15, Pos. 9) ins Spiel gebracht. Vergleichbar mit traditionellen familialen Bindungen wird der Hilfe dabei auch kaum eine persönliche Abgrenzungsmöglichkeit zugestanden. Sie hat rund um die Uhr unbeschränkt für vielfältige Aufgaben zur Verfügung zu stehen. Gelegentlich wird geradezu eingeräumt, dass hier Erwartungen im Spiel sind, die die Angehörigen selbst in ihrer Rolle als Familienmitglieder nicht (mehr) erfüllen können oder wollen. Ihren sprachlichen Ausdruck findet diese familiäre Vereinnahmung der Live-in-Hilfe beispielhaft in der besitzergreifenden Ansprache als „unsere Maria“ (Alz 2.7, Pos. 10), die nach erfüllter Aufgabe konsequenterweise auch innerhalb der Familie weitervermittelt wird.

Derartige Erwartungen an die Live-in-Hilfe als Familienmitglied bergen hohes Konflikt- und Enttäuschungspotenzial. Vielfach nehmen Live-in-Hilfen im Leben der zu versorgenden Menschen eine wichtige Stellung ein, die aufgrund räumlicher Nähe vertrauter erscheinen kann als die der in einem anderen Haushalt lebenden Angehörigen selbst. Dabei sollen sie sich jedoch idealerweise zugleich in die bestehenden hierarchischen Strukturen und Machtverhältnisse des familiären Versorgungssettings einfügen. Als Negativbeispiel berichtet eine Angehörige davon, wie Live-in-Hilfen „die Haushaltsführung wie ein Haushaltsvorstand versahen“ und beschreibt deren Haltung mit: „Ich Boss. Du nichts.“ (Alz, anonym, Pos. 13). Tatsächlich können die mit ihrem Einzug einhergehenden Verschiebungen im innerfamiliären Beziehungsgefüge auch als Ausdruck intriganter Manipulation oder gar strategisch motivierter Verdrängung der Familienangehörigen empfunden werden: Der Vater einer:s Nutzer:in sei „abgeschirmt“ worden, die Hilfe habe sich „unentbehrlich“ gemacht, während sich die Nutzer:in selbst in der Rolle der „feindseligen Geldabzockerin“ wiederfand (Alz, anonym, Pos. 13).

Die besondere emotionale Nähe und persönliche Beziehung zwischen Live-in-Hilfe und zu pflegender Person wird vonseiten der Angehörigen vor allem beschworen, wenn es um Erwartungen hinsichtlich des Einsatzes und der Qualität der Versorgung geht. Legt die Hilfe am familiären Anspruch gemessen einen Mangel an emotionaler Emphase oder persönlicher Loyalität und Verbundenheit an den Tag oder lässt gar eigennützige, etwa monetäre Motive erkennen, erscheint die Enttäuschung vorprogrammiert. Das Verfolgen monetärer Interessen wird der Live-in-Hilfe in der Erwartung einer selbstlosen Aufopferung als quasi-Familienmitglied als unmoralisch vorgeworfen. Gleichzeitig wird in den analysierten Onlineforen eine monetär begründete Motivation zur Arbeit als Live-in-Hilfe als Ausdruck und Ausweis ihres freien Willens als Vertragspartner:in und Dienstleistungserbringer:in angesehen. Der in anderen Argumentationszusammenhängen valide Verweis auf vertragliche Vereinbarungen, professionelle Distanz oder persönliche Grenzen scheint jedoch im Kontext familiärer Rollenerwartungen nicht länger zu zählen. So erweist sich die Stellung der Live-in-Hilfe auch in dieser Hinsicht als prekär, ambivalent und hin- und hergerissen zwischen konfligierenden Ansprüchen und gelegentlich unvereinbaren Rollenerwartungen.

## Schluss: Enttäuschungen vorprogrammiert? Das Spannungsfeld moralischer Perspektiven auf Live-in-Versorgung

In der Gesamtschau der in den Onlineforen von Angehörigen geäußerten Erwartungen gegenüber Live-in-Hilfen in der häuslichen Versorgung von Menschen mit Demenz konnten typologisierend vier Rollenbilder identifiziert werden: erstens die Rolle der:des Dienstleistungserbringer:in, die im Rahmen vertraglicher Abmachungen als identitätslose und potenziell austauschbare Arbeitskraft hinsichtlich des Umfangs und der Qualität ihrer Leistungen beurteilt wird; zweitens die der Pflegekraft, die hohe professionelle Kompetenz im Umgang mit kranken alten Menschen aufweist und unter Bezugnahme auf professionsethische Prinzipien bewertet wird; drittens die der:des moralischen Akteur:in, die Tugenden der Ehrlichkeit, Verlässlichkeit, Rücksichtnahme und Höflichkeit an den Tag legen soll, und viertens die des Familienmitglieds, von dem erwartet wird, dass es sich in so umfassender Weise dem Wohl der zu versorgenden Person widmet, wie es allenfalls im Kontext filialer oder elterlicher Verpflichtungen vorausgesetzt werden könnte.

Aus ethischer Perspektive erscheinen die unterschiedlichen Erwartungen an die Live-in-Hilfe schon jeweils für sich betrachtet vielfach problematisch. So verweisen die mit der Dienstleistungsrolle verknüpften Erwartungen der persönlichen Indifferenz und beliebigen Austauschbarkeit auf ein anonymes und potenziell entfremdetes, objektivierendes und instrumentalisierendes Arbeitsverhältnis. Die hohen Anforderungen hinsichtlich der professionellen Qualität von Live-in-Versorgung, die sich in der Rolle der Pflegekraft verdichten, stehen in keinem angemessenen Verhältnis zu ihrer vergleichsweise niedrigen Bezahlung sowie den oft kaum klar geregelten Arbeitszeiten und -bedingungen. Die Erwartungen hinsichtlich Tugendhaftigkeit, die sich auf die Live-in-Hilfe als moralische Akteurin richten, bewegen sich angesichts der häufig prekären Lebens- und Arbeitssituation der betreffenden Personen nicht selten in einem Spannungsfeld von verständnisloser Überforderung und herablassender Nachsicht. Die Erwartungen der intrinsischen, emotionalen Motivation und scheinbar grenzenlosen persönlichen Aufopferung sowie zeitlichen Verfügbarkeit der Live-in-Hilfe als Familienmitglied unterstreichen schließlich noch einmal den transgressiven, vereinnahmenden und tendenziell ausbeuterischen Charakter eines rechtlich unzulänglich definierten Arbeitsverhältnisses.

Erschwerend kommt hinzu, dass diese bereits in sich problematischen Erwartungstypen vielfach auch kaum miteinander zu vereinbaren sind, in den Forumsdiskussionen de facto aber immer wieder gleichzeitig auftreten, sich überschneiden und ineinander übergehen. Einerseits soll die Live-in-Hilfe mit der inneren Unbeteiligtheit und Geschäftsmäßigkeit der:des angeheuerten Dienstleistungserbringer:in agieren, andererseits aber im Umgang mit der ihr anvertrauten Person mit Demenz in ihrer privaten Häuslichkeit eine besondere moralische Sensibilität und Verantwortungsbereitschaft an den Tag legen. Einerseits soll sie den hohen Anforderungen und emotionalen Belastungen der Demenzversorgung mit der professionellen Distanz und Kompetenz der ausgebildeten Pflegekraft begegnen, sich andererseits aber zugewandt und aufopferungsvoll um die zu pflegende Person kümmern wie eine nahe Angehörige. Dieses Oszillieren spiegelt grundlegende moralische Unklarheiten, Spannungen und Konflikte wider, die die Live-in-Versorgung als einen rechtlich unzulänglich regulierten und gesellschaftlich nicht konsentierten Raum bestimmen. Letzten Endes tritt hier eine konstitutive Überforderung der Live-in-Position zu Tage, an die sich eine Vielzahl unterschiedlicher, teilweise divergierender und mitunter widersprüchlicher Erwartungen richten.

Allerdings können diese Probleme und Widersprüche aufseiten der Angehörigen durch die verschwimmenden Grenzen, fließenden Übergänge und dynamischen Wechsel der verschiedenen Rollenerwartungen und dahinterstehenden normativen Bezugsrahmen immer wieder auch argumentativ überspielt und damit letztlich latent gehalten werden. Dies zeigt sich im Material z. B. daran, wie Erwartungen an die Live-in-Hilfe als bezahlte:r Dienstleistungserbringer:in durch die Bezugnahme auf den familiären und persönlichen Kontext des Versorgungsverhältnisses relativiert und verschleiert werden. An der Schnittstelle zwischen dem informellen familiären Versorgungssetting einerseits und durch Vermittlungsagenturen propagierter formalisierter und kommodifizierter Versorgung andererseits kommen unterschiedliche interessengebundene und -geleitete Darstellungen der Wirklichkeit von Live-in-Versorgung zum Tragen, z. B. einerseits der Wunsch nach einem angemessenen Ersatz für die zugewandte und aufopferungsvolle Versorgung durch eine:n nahe:n Angehörige:n und andererseits das Interesse an einer sowohl kostengünstigen als auch professionellen Pflege im eigenen Zuhause. So spiegeln sich in den Rollenkonflikten der Live-in-Hilfe letzten Endes auch strukturelle Widersprüche einer Gesellschaft, deren moralische Ideale der Sorge für hilfsbedürftige Menschen sich im Rahmen ihrer eigenen sozialpolitischen Regulationen und ökonomischen Funktionszusammenhänge kaum angemessen verwirklichen lässt.

Die vorgestellten Ergebnisse ergänzen die bisherige Forschung zu Konflikten im Live-in-Kontext. Die vorliegenden sozialwissenschaftlichen Studien nehmen vorrangig die Live-in-Hilfen selbst in den Blick und beleuchten die strukturellen sozialen Probleme ihrer Situation, etwa die transnationalen Situierungen zwischen Heimat und Arbeitsplatz (z. B. Lutz und Palenga-Möllenbeck 2011; Schilliger 2014) und die teilweise ausbeuterischen und rechtlich unzulässigen Arbeitsbedingungen (z. B. Emunds 2016; Bucher 2018). In ihren Untersuchungen der Angehörigenperspektiven heben Horn und Kolleg:innen (Horn et al. 2019) die Schwierigkeiten der Angehörigen hervor, angemessene Rechtfertigungen für diese strukturellen sozialen Probleme hinsichtlich Arbeitsbedingungen und Bezahlung zu finden. Rossow vertieft den Blick auf die Perspektiven von Angehörigen im Live-in-Arrangement (Rossow 2021). Zunächst benennt sie die Abwendung des drohenden Autonomieverlusts der Angehörigen durch den entstehenden Versorgungsaufwand als zentrale Motivation zur Inanspruchnahme von Live-in-Versorgung (Rossow 2021, S. 153 ff.). Zudem konstatiert sie ein „Intimitätsparadoxon“: Die immer wieder enttäuschten Erwartungen an die Herstellung eines intimen Versorgungsverhältnisses durch die eigentlich fremde Live-in-Hilfe seien Ausdruck des Konflikts zwischen den widersprüchlichen normativen Bezugsrahmen der Tauschakte des Marktes und der Reziprozitätsnormen des Versorgungssettings (Rossow 2021, S. 200 ff.). Unsere Typologie erlaubt es, diese Ergebnisse aus einer dezidiert ethischen Perspektive weiter zu differenzieren. Insbesondere veranschaulicht sie die Vielfalt der moralischen Gesichtspunkte, die den von Rossow herausgearbeiteten Schemata der Bewertung der Live-in-Arbeit („idealisierend“, „wertschätzend“, „zur Kenntnis nehmend“ und „abwertend“) zugrunde liegen (Rossow 2021, S. 194 ff.). So zeigt sich, dass über das Spannungsfeld der beiden normativen Bezugsrahmen des Marktes und der intimen Häuslichkeit hinaus unterschiedliche und konfligierende Rollenbilder und entsprechende moralische Bewertungsmaßstäbe zum Tragen kommen können. Unsere Typologie verdeutlicht, dass die Bewertung von Live-in-Arbeit vor dem Hintergrund von Erwartungen erfolgen kann, die sich aus allen vier herausgearbeiteten Rollenbildern speisen. Wenn Angehörige etwa die Live-in-Hilfe als untätig abwerten und sich deswegen bei ihrer Vermittlungsagentur beschweren (Rossow 2021, S. 196), kann dies gleichzeitig auf die Idealbilder einer vertragserfüllenden Dienstleistungserbringer:in, einer fürsorglichen und verantwortungsbewussten Pflegekraft, einer tugendhaften, fleißigen und verlässlichen moralische:n Akteur:in, und letztendlich eines:r aufopferungsvollen und selbstlosen Familienangehörigen verweisen. So treten in unserer eigenen Untersuchung die komplexen moralischen Konflikte im Mikrosetting der Versorgungskonstellation bestehend aus Mensch mit Demenz, Angehörigen und Live-in-Hilfe noch deutlicher in den Vordergrund. Dabei erlaubt die ethische Analyseperspektive eine eingehendere Betrachtung der vielfältigen Erwartungen an Live-in-Versorgung im Lichte ihrer unterschiedlichen normativen Bezugsrahmen und deren innerer Kohärenz und wechselseitiger Vereinbarkeit. Auf diese Weise wird eine vertiefende und differenziertere kritische Auseinandersetzung mit den im Rahmen der Live-in-Versorgung erhobenen Ansprüchen im Hinblick auf ihre normative Berechtigung möglich.

Allerdings weist auch unsere Studie gewisse methodische Limitationen auf. Zwar hat die Analyse von Beiträgen in Online-Foren den Vorteil, dass Probleme erfasst werden können, die bspw. in Interviews aufgrund eines Bias der sozialen Erwünschtheit nicht genannt werden würden. Zugleich können die sozialen Dynamiken in Online-Foren aber ihrerseits zu Verzerrungen führen. So können beispielsweise innerhalb eines Threads Mehrheitsmeinungen tendenziell verstärkt und abweichende Meinungen eher zum Verstummen gebracht werden (Woong Yun und Park 2011). Deshalb besteht die Gefahr, dass es in bestimmten Threads oder Foren zur einseitig verzerrten Repräsentation negativer bzw. positiver Erfahrungen oder Bewertungen kommt. Hier wäre die Onlineforschung zu moralischen Perspektiven auf Live-in-Versorgung entsprechend durch gezielte Einzelinterviews zu ergänzen und zu kontrastieren. Und schließlich hat sich unsere Untersuchung der moralischen Probleme von Live-in-Versorgung für Menschen mit Demenz zunächst auf die Perspektiven von Angehörigen konzentriert. Weiterführende Studien müssten auch die moralischen Perspektiven und Ansprüche der anderen Beteiligten des Mikrosettings in die ethische Analyse einbeziehen, um zu einem umfassenderen und ausgewogeneren Bild zu gelangen.

Eine solche eingehendere ethische Analyse und Erörterung der Erwartungen an Live-in-Versorgung verspricht auf mindestens drei Ebenen notwendige und sinnvolle Beiträge zur Verbesserung der Ausgangslage: Auf der Mikroebene kann sie im Sinne eines Beratungsangebots, beispielsweise durch Pflegestützpunkte, zur frühzeitigen Klärung unrealistischer, problematischer und illegitimer Ansprüche, zur vorausschauenden Planung und Einbettung von Live-in-Versorgung in ein umfassenderes Netz familialer und professioneller Sorgeverantwortlichkeiten und damit zur vorsorglichen Vermeidung und Entschärfung von Konfliktpotenzialen beitragen. Diese präventive Aufgabe kann überdies auf der Mesoebene der Vermittlungsagenturen durch Regulierungen unterstützt werden, die zu einem verantwortungsvollen Informationsangebot und Erwartungsmanagement gegenüber den Angehörigen und allen anderen an den transnationalen Versorgungsarrangements beteiligten Akteur:innen verpflichten. Und schließlich hat die ethische Analyse auch Konsequenzen für den gesellschaftlichen und politischen Umgang mit Live-in-Versorgung, indem sie die Existenz unterschiedlicher Interessen in der Live-in-Versorgung von Menschen mit Demenz aufdeckt und eine offene Verständigung über ihre jeweilige normative Berechtigung und Gewichtung unterstützt. Eine Legalisierung „nach dem österreichischen Modell“ (Emunds et al. 2021, S. 7), wie sie unter anderem vom (Lobby‑)Verband für häusliche Betreuung und Pflege (VHBP) gefordert (VHBP e. V. 2022) und sowohl vom Eckpunktepapier des Bundesministeriums für Gesundheit (BMG 2020) als auch vom Koalitionsvertrag der Ampelparteien angedeutet wird, mag zwar die Last der moralischen Verantwortung für die Inanspruchnahme von auf transnationalen Ungleichheiten und vergeschlechtlichten Care-Chains basierender Versorgung von den Schultern der Angehörigen nehmen und diese Versorgungsform als Ausdruck eines vermeintlich gesamtgesellschaftlichen Willens legitimieren. Sie wird jedoch nicht die moralischen Konflikte der verschiedenen Anliegen im Mikrosetting lösen, die auf die strukturellen Widersprüche einer häuslichen Versorgung von hilfsbedürftigen Menschen (mit Demenz) zurückzuführen sind, deren Zeit- und Arbeitsaufwand nicht nur pflegende Angehörige überfordert, sondern auch jedes reguläre Erwerbsarbeitsverhältnis sprengt (Rossow und Leiber 2022). Ganz in diesem Sinne bezeichnet letztlich auch das Landesarbeitsgericht Berlin-Brandenburg in seiner Urteilsbegründung zum eingangs genannten Fall die Berufung der Beklagten auf die vertraglich vereinbarte Arbeitszeit von 30 Stunden die Woche als „unzulässig, weil widersprüchlich“ (LArbG Berlin-Brandenburg 21. Kammer 2020, S. 98).
